# An architectonic type principle integrates macroscopic cortico-cortical connections with intrinsic cortical circuits of the primate brain

**DOI:** 10.1162/netn_a_00100

**Published:** 2019-09-01

**Authors:** Claus C. Hilgetag, Sarah F. Beul, Sacha J. van Albada, Alexandros Goulas

**Affiliations:** Institute of Computational Neuroscience, University Medical Center Eppendorf, Hamburg University, Germany; Department of Health Sciences, Boston University, Boston, MA, USA; Institute of Computational Neuroscience, University Medical Center Eppendorf, Hamburg University, Germany; Institute of Neuroscience and Medicine (INM-6), Institute for Advanced Simulation (IAS-6), and JARA-Institute of Brain Structure-Function Relationships (INM-10), Jülich Research Centre, Germany; Institute of Computational Neuroscience, University Medical Center Eppendorf, Hamburg University, Germany

**Keywords:** Cortical connectome, Wiring principles, Cortical structural gradients, Cytoarchitecture

## Abstract

The connections linking neurons within and between cerebral cortical areas form a multiscale network for communication. We review recent work relating essential features of cortico-cortical connections, such as their existence and laminar origins and terminations, to fundamental structural parameters of cortical areas, such as their distance, similarity in cytoarchitecture, defined by lamination or neuronal density, and other macroscopic and microscopic structural features. These analyses demonstrate the presence of an architectonic type principle. Across species and cortices, the essential features of cortico-cortical connections vary consistently and strongly with the cytoarchitectonic similarity of cortical areas. By contrast, in multivariate analyses such relations were not found consistently for distance, similarity of cortical thickness, or cellular morphology. Gradients of laminar cortical differentiation, as reflected in overall neuronal density, also correspond to regional variations of cellular features, forming a spatially ordered natural axis of concerted architectonic and connectional changes across the cortical sheet. The robustness of findings across mammalian brains allows cross-species predictions of the existence and laminar patterns of projections, including estimates for the human brain that are not yet available experimentally. The architectonic type principle integrates cortical connectivity and architecture across scales, with implications for computational explorations of cortical physiology and developmental mechanisms.

## Author Summary

The mammalian cortex possesses multiple dimensions of organization, for instance, the connectional and the cytoarchitectonic dimension. Are there principles that link the different dimensions? Here we review an architectonic type principle that links cytoarchitectonic aspects of the cerebral cortex, such as neuron density or morphology across the cortical layers, to large-scale interregional cortical connection patterns. The reviewed findings highlight the existence of a natural axis of spatially ordered, concerted changes of multiple architectonic, connectional, and functional features stretching from less to more differentiated cortical areas. This framework comprises species-general, but also species-specific, principles of the organization of the mammalian, and particularly the primate, cerebral cortex and highlights potential developmental underpinnings as well as functional ramifications of such principles.

## SEARCHING FOR PRINCIPLES OF CORTICAL CONNECTIVITY

### Why Search for Principles of Brain Organization

The wiring of the cerebral cortex appears highly structured, but its precise organization is overwhelmingly difficult to discern. In particular, the large number of neural elements and the vast number of intricate interactions among them obscure potential regularities. A similar level of complexity exists in other aspects of brain organization, such as the diversity and distribution of different cell types (Cembrowski & Menon, 2018; Molyneaux, Arlotta, Menezes, & Macklis, 2007), the arrangement of neurotransmitters and receptors (Palomero-Gallagher & Zilles, 2017; Zilles & Palomero-Gallagher, 2017), and diverse morphological features at the micro- (cellular) (Elston, 2002, 2003) and macro- (regional) scale. Thus, at first sight, it appears almost impossible to integrate the many different dimensions of brain organization. Fortunately, however, some of these aspects are intrinsically related through fundamental organizational principles, substantially reducing the dimensionality of the problem. Thus, underlying principles of cortical wiring and architecture curb the complexity of cortical organization. Moreover, the discovery of such integrative principles, linking the different dimensions of cortical organization, may hint at central mechanisms of brain evolution and development and may facilitate the understanding of brain function.

**Figure 1.**
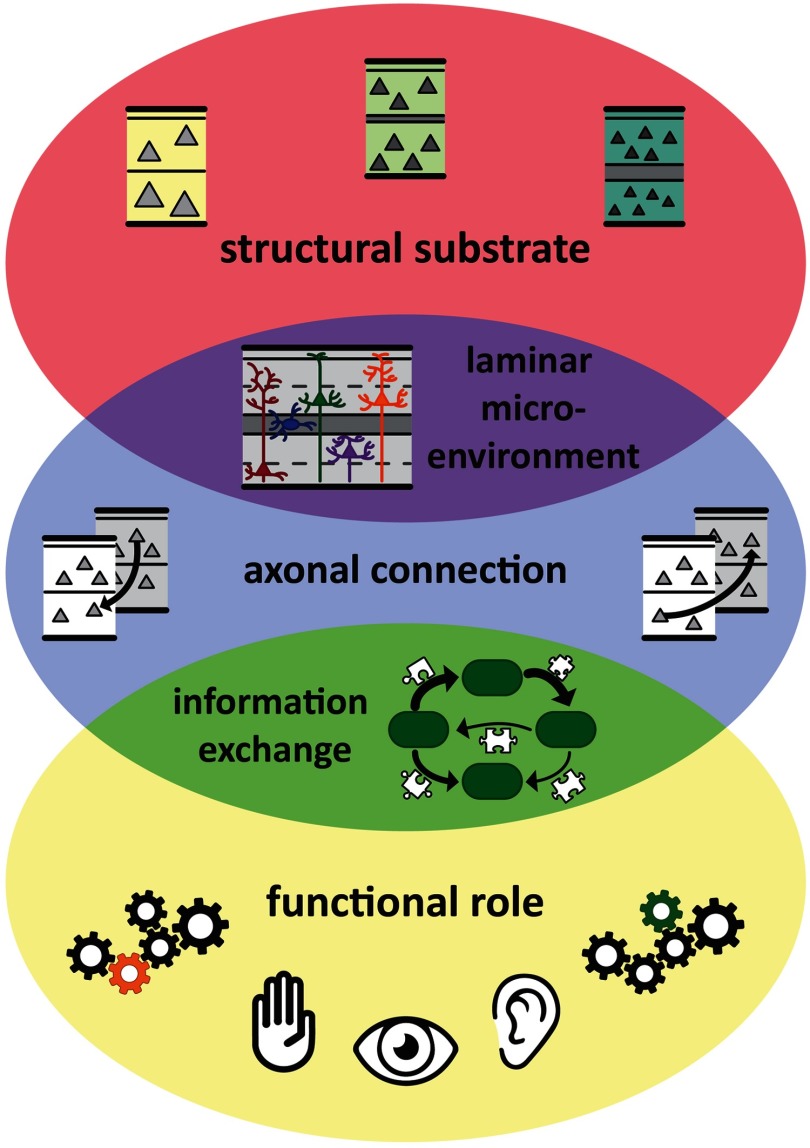
Principles of cortical wiring integrate regularities of cortical architecture, connectivity, and function through mechanistic explanations. Connections create functions of brain areas, and functional interactions among areas, from the structural substrate of the brain, and specifically the cortical sheet. In particular, areas are linked through connections which have a laminar composition that is appropriate for the laminar microenvironment within the respective areas as well as the type of information exchange between these areas. Thus, local cortical architecture, the connection features of a cortical area, and an area’s functional role within the cortical network are tightly intertwined. Adapted from Beul and Hilgetag (2019).

Motivated by the wish to integrate different dimensions of brain organization and the hope to understand mechanisms of development and evolution that produce the complex neural substrate, several groups including ours have been striving to identify regularities and principles in the organization of brain connectivity, particularly the macroscopic interareal connections of the mammalian cerebral cortex, frequently guided by a cross-species mammalian perspective (Goulas, Majka, Rosa, & Hilgetag, 2019; Goulas, Zilles, & Hilgetag, 2018; van den Heuvel, Bullmore, & Sporns, 2016). In this context they have also investigated the relation of connection features to other structural aspects of the brain. Note that here we reserve the term “principle” for regularities that can be supported by mechanistic explanations of their occurrence. In particular, principles of cortical wiring may be explained by mechanisms of brain development and plasticity, and may allow the integration of cortical structure, connectivity, and function (Figure 1).

While there are many possible aspects of wiring that could be addressed, a helpful initial goal is to explain basic features of cortical connections, such as the existence and density of connections as well as the patterns of laminar origins and terminations of projections. Another such feature would be the direction of projections (Kale, Zalesky, & Gollo, 2018). Thus, in the present review, we do not primarily focus on high-order organizational features of cortical brain networks, such as, for example, their rich-club topology, which are worthwhile features to explain in their own right, but may in fact arise from the more basic aspects of brain network organization (Rubinov, 2016).

### Physical Embedding of Connectivity and Minimal Wiring

One well-established and intuitive idea is that brain connectivity is shaped by the physical embedding of the brain in space (Henderson & Robinson, 2011; Roberts et al., 2016), which constrains the development of neural connections (Kaiser & Hilgetag, 2004; Kaiser, Hilgetag, & van Ooyen, 2009). The idea of a strong influence of the spatial layout on the organization of connections is also related to the engineering-inspired concept of minimal wiring (Ramón y Cajal, 1899), which suggests that the length and volume of wiring reflects a substantial cost in brain development and function and should thus be as small as possible (reviewed in Sterling & Laughlin, 2015). While numerous studies have demonstrated that wiring economy indeed appears to affect the organization of brain connectivity, as reviewed in Bullmore and Sporns (2012), it is also clear that minimal wiring is not the only structural or functional constraint on brain organization (Kaiser & Hilgetag, 2006; Roberts et al., 2016; Rubinov, 2016). Instead, the brain is subject to multiple structural and functional evolutionary constraints that may be partly antagonistic (Chen, Wang, Hilgetag, & Zhou, 2013, 2017), as well as constraints imposed by the evolutionary history and dynamic stability of the nervous system (Gollo et al., 2018).

### Embedding of Cortical Connectivity in Brain Architecture

One alternative perspective on brain organization also has a long tradition in brain research, particularly in the investigation of the cerebral cortex. Classical neuroanatomists, such as Brodmann, the Vogts, or von Economo and Koskinas, used regional variations in architectonic features of the brain, such as neuronal density or thickness of cortical layers, in order to parcellate and characterize brain regions. This work was founded on the central tenet of biology, already recognized by Aristotle^1^ (350 AD), that differences in biological function should vary with differences of structure. Thus, the architectonic differences of cortical areas were used to delineate structural parcels that may also operate as specialized functional units.

Such anatomical approaches observed that there may be systematic variations, in terms of spatially defined gradients of brain architecture, as expressed by the differential density of neural populations across the cortical layers. In particular, von Economo and Koskinas categorized areas into so-called cortical types (von Economo, 1927; von Economo & Koskinas, 1925), and Sanides (1962) linked gradients of cortical types to their evolutionary origins. It was also suggested that such architectonic gradients shape basic features of connectivity (Pandya & Sanides, 1973). In particular, in her “structural model of connections” (García-Cabezas, Zikopoulos, & Barbas, 2019), H. Barbas proposed that laminar terminations and origin patterns of prefrontal cortical areas in the primate brain are directly linked to the relative differences in the laminar differentiation and organization of cortical areas (Barbas, 1986; Barbas & Rempel-Clower, 1997).

### Relations of Connectivity to Other Aspects of Brain Structure

Several further factors that may be related to connectivity have been tested. These factors include covariation of the overall cortical thickness of different regions as a proxy for connectivity (He, Chen, & Evans, 2007; Lerch et al., 2006) or MR-based measurements (Seidlitz et al., 2018) indicating structural similarity. However, the actual relation of these “morphological networks” to structural or functional connectivity warrants further investigation, as these measures do not map directly onto each other (Reid et al., 2016), and, therefore, “connectivity” in the different contexts is used with different meanings and potentially reflects different neurobiological aspects.

Patterns of gene expression may also be directly linked to connectivity (Fulcher & Fornito, 2016) or structural covariation (Romero-Garcia et al., 2018). Moreover, several studies have explored which cellular properties are related to connectivity (Scholtens, Schmidt, de Reus, & van den Heuvel, 2014; van den Heuvel, Scholtens, Barrett, Hilgetag, & de Reus, 2015). In particular, these studies found a relation of the cell size of layer III pyramidal cells in different cortical areas with features of connectivity, such as the number of connections that these areas form. Generally, while there are findings that several architectonic features at the macroscale (such as cortical thickness) or microscale (cell size, density) may be related to cortico-cortical connectivity, the systematic relations among these features and connectivity are still unclear. In any case, the potential linkages among these measures necessitate the joint multivariate analysis of as many available features as possible.

Based on these potential correlates of connectivity, our goal has been to systematically test different concepts of cortical connectivity organization. Thus, we performed a series of studies in various cortical regions and across mammalian species, which are summarized in the following sections. We used a wide range of variables to investigate the embedding of fundamental features of connectivity in brain space or brain architecture. In particular, we used Euclidean and geodesic distance as well as border distance as measures of spatial separation and characterized essential features of cortical architecture categorically as well as quantitatively. In this context, we focused on a fundamental characterization of cortical architecture as reflected in cortical type. Cortical type is a classical, comprehensive characterization of cortical parcels and comprises the apparent density of cellular populations across the cortical layers (Hilgetag, Medalla, Beul, & Barbas, 2016). A simple quantitative proxy of cortical type is neuronal density, measured across all cortical layers (Medalla & Barbas, 2006). It is already known that the classical measure of neuronal density is the most characteristic measure for identifying cortical areas (Dombrowski, Hilgetag, & Barbas, 2001). In addition, we considered further morphological and cellular markers of the structural organization of cortical areas, such as cortical thickness, and cellular features, such as layer III pyramidal cell soma cross section, dendritic synapse count, dendritic synapse density, and dendritic tree size.

## THE ARCHITECTONIC TYPE PRINCIPLE UNDERLIES THE CONNECTIVITY OF THE PRIMATE CONNECTOME

In order to assess the relations between essential features of cortical connections and cortical architecture, we investigated a comprehensive, quantitative compilation of connectivity data (a *connectome*) for cortico-cortical connections of the macaque monkey brain (Markov, Ercsey-Ravasz, et al., 2014; Markov, Vezoli, et al., 2014) together with quantitative measures of various aspects of cortical structure. Present and absent pathways differ in terms of the features of the potentially connected cortical areas. Areas linked by a connection are more similar in terms of neuronal density and cortical thickness, and in terms of cellular morphological features such as spine density and dendritic arborization. Moreover, connected areas are also spatially closer to each other than unconnected areas (Beul & Hilgetag, 2019). However, all of these structural features are related to each other (cf. Figure 4). Therefore, multivariate analyses are required to disentangle the essential structural contributions to connectivity features. Using such an analysis (multivariate regression), we found that the most fundamental contributions came from just two factors, neuronal density and distance, where in fact neuronal density was the cortical dimension that was more consistently related to the existence and laminar origin of connections (Beul & Hilgetag, 2019).

These insights can be used to predict connections by regression based on the proximity and architectonic similarity of cortical areas. This approach allows one to assess the individual and combined contributions of the different structural factors to explaining the existence of cortical connections. The measures showed that architectonic similarity as well as distance were individually strongly predictive of connections, while similarity of cortical thickness was not. However, the best prediction performance was achieved by combining architectonic similarity with distance, leading to high classification accuracy (Beul, Barbas, & Hilgetag, 2017).

Notably, the number of connections of an area (the area’s degree, in graph-theoretical terms) was found to be inversely correlated to the area’s type or neuronal density, with less dense (low-type) areas having more connections than dense (high-type) areas (Beul et al., 2017). Furthermore, core or rich-club areas (Ercsey-Ravasz et al., 2013; Harriger, van den Heuvel, & Sporns, 2012) are of low type; that is, they possess low neuronal density.

Another essential feature of cortico-cortical projections is the pattern of their origins and terminations in the cortical layers, which shapes spectral channels of interareal communication (Bastos et al., 2015) and is a central feature in theories of brain function such as predictive coding (Bastos et al., 2012). The only factor that was significantly correlated with the patterns of laminar origins of primate cortico-cortical projections (Markov, Vezoli, et al., 2014) was the relative neuronal density of the source versus the target area of the projection (Beul & Hilgetag, 2019). Larger positive differences in neuron density from connection source to connection target were associated with projections mainly originating from upper cortical layers, whereas negative neuron density differences were associated with projection origins in deep cortical layers. None of the other tested parameters showed a systematic correlation with the laminar projection patterns. Thus, the cytoarchitectonic gradients of the cerebral cortex, reflected in graded neuronal density differences, are the fundamental dimensions across which systematic shifts in the laminar origin of connections occur (Barbas, 1986; Barbas & Rempel-Clower, 1997; Beul & Hilgetag, 2019; Goulas et al., 2018).

## EVIDENCE FOR THE ARCHITECTONIC TYPE PRINCIPLE ACROSS CORTICES AND SPECIES

### Cat Cortical Connectome

Analyses of further datasets widely confirm the findings from the primate connectome. In particular, an analysis of the cat cortico-cortical connectome (Scannell, Blakemore, & Young, 1995) yielded very similar results (Beul, Grant, & Hilgetag, 2015). In a multivariate linear discriminant analysis (LDA), border distance as well as architectonic type difference were found to be associated with the presence or absence of connections, type difference more so than distance, but the best classification accuracy was achieved by combining the two factors.

As in the primate, the type of an area was inversely associated with the number of connections formed by the area, with low-type areas, including the core of the cat cortex (Zamora-López, Zhou, & Kurths, 2010), forming more connections.

Type differences were also significantly associated with the laminar projection patterns of the cortico-cortical connections, such that projections from a higher type to a lower type area formed forward pathways, while projections from the lower type to a higher type area formed feedback projections (Beul et al., 2015; Hilgetag & Grant, 2010), once again highlighting the cytoarchitectonic gradients of the cortex as a fundamental dimension across which systematic changes of the laminar origin of connections manifest.

### Mouse Cortical Macroconnectome

As in the primate and the cat, the existence of cortico-cortical connections in the mouse was associated with spatial proximity as well as similarity of cortical type of the potentially connected areas (Goulas, Uylings, & Hilgetag, 2017). Interestingly, distance appeared to contribute more strongly to the prediction of ipsilateral projections, while architectonic similarity contributed more strongly for contralateral projections. Tests of the relationship between cortical types and the laminar projection patterns in the mouse await the full release of such projection information. Nonetheless, the architectonic type principle and its accentuation across the spectrum of mammalian cortical architecture (Goulas et al., 2018) allow predictions of laminar projection patterns in presently untested connectomes. Specifically, for rodents, who have architectonically less well-differentiated upper cortical layers than species such as the cat or macaque monkey, we predict that the laminar origin of connections will be less varied across the cortical sheet, with most projections having a bilaminar origin or originating from the deep cortical layers (cf. Hilgetag & Grant, 2010).

### Findings for Further Connectivity Data

In addition to the studies already described above, there is a wealth of evidence supporting the architectonic type principle across different cortical regions and across different species. Originally, Barbas (1986) demonstrated the correlation between the laminar origin patterns of projections and the cortical type of the projection origin for projections to the prefrontal cortex in the primate brain. These findings were later extended to terminations of prefrontal connections (Barbas & Rempel-Clower, 1997) and connections of the prefrontal cortex with other lobes. Moreover, the architectonic type principle also applies to the laminar origins of projections to the amygdala (Ghashghaei, Hilgetag, & Barbas, 2007), and to the laminar patterns and existence of cortico-cortical connections with the contralateral hemisphere in the primate (Barbas, Hilgetag, Saha, Dermon, & Suski, 2005). In a study of the laminar patterns of parietal-prefrontal projections, Medalla and Barbas (2006) demonstrated that overall neuronal density can be used as a metric proxy of cortical type, and that this variable also explained small variations of the laminar patterns. Using this proxy, Schmidt, Bakker, Hilgetag, Diesmann, and van Albada (2018) predicted laminar origin patterns in macaque vision-related cortex. The analysis further showed laminar termination patterns from the CoCoMac database (Bakker, Wachtler, & Diesmann, 2012) to relate to the laminar origin patterns, consistent with the classic work of Felleman and Van Essen (1991). Thus, differences in neuronal density or architectonic type are also predictive of laminar termination patterns among the vision-related areas of macaque cortex (Hilgetag et al., 2016).

In addition, the architectonic type principle was observed for laminar origin patterns of extrastriate projections in the cat visual cortex (Hilgetag & Grant, 2010), as well as for the existence and absence of connections and laminar origin patterns of the entire cat cortical connectome (see above). Thus, there is widespread evidence across mammalian species and different types of cortex of the relation of architectonic differentiation with essential features of macroscopic cortical connectivity, giving rise to the hypothesis that this association may also be present in the human brain (Goulas et al., 2016; Solari & Stoner, 2011).

**Figure 2.**
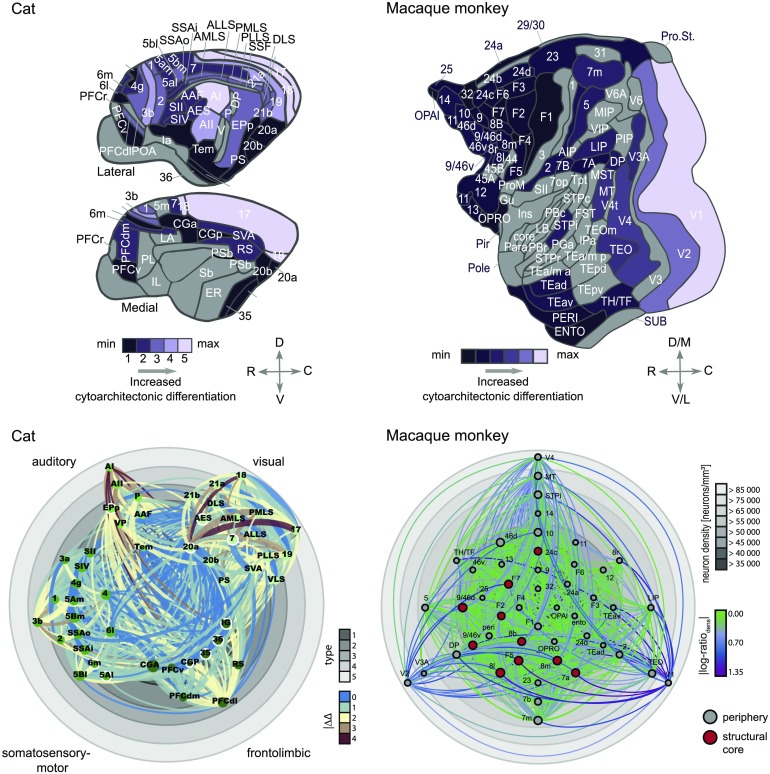
Architectonic type principle across species. Upper panels: Maps of the cat and macaque cortex, indicating the variation of architectonic differentiation across the cortex of these two species. Differentiation is represented as architectonic type in the cat cortex and as neuron density in the macaque cortex. Lower panels: Visualization of cortico-cortical connections in the cat and macaque cortex. Cat connections are shown as collated in Scannell et al. (1995); macaque connections are shown as published by Markov, Ercsey-Ravasz, et al. (2014). Gray rings correspond to degree of architectonic differentiation (determined as cortical type for the cat and by neuron density for the macaque) and cortical areas are placed accordingly, with differentiation increasing from center to periphery. Projections are color coded according to the difference in architectonic differentiation between connected areas. Node sizes indicate the areas’ degree (that is, the number of connections associated with them). For the cat cortex, ordinal projection strength (sparse, intermediate, or dense) is coded by increasing projection width and nodes are grouped and color coded according to anatomical modules as indicated. Hub-module areas, as classified by Zamora-López et al. (2010) in the cat and Ercsey-Ravasz et al. (2013) in the macaque, are marked by a white outline or red fill, respectively. Panels adapted from Beul et al. (2017, 2015).

These findings may be summarized in cortical wiring diagrams (Figure 2) that show the arrangement of cortical areas and their connections according to cortical types, with the most highly differentiated areas on the outside and more poorly differentiated areas on the inside of the diagram.

**Figure 3.**
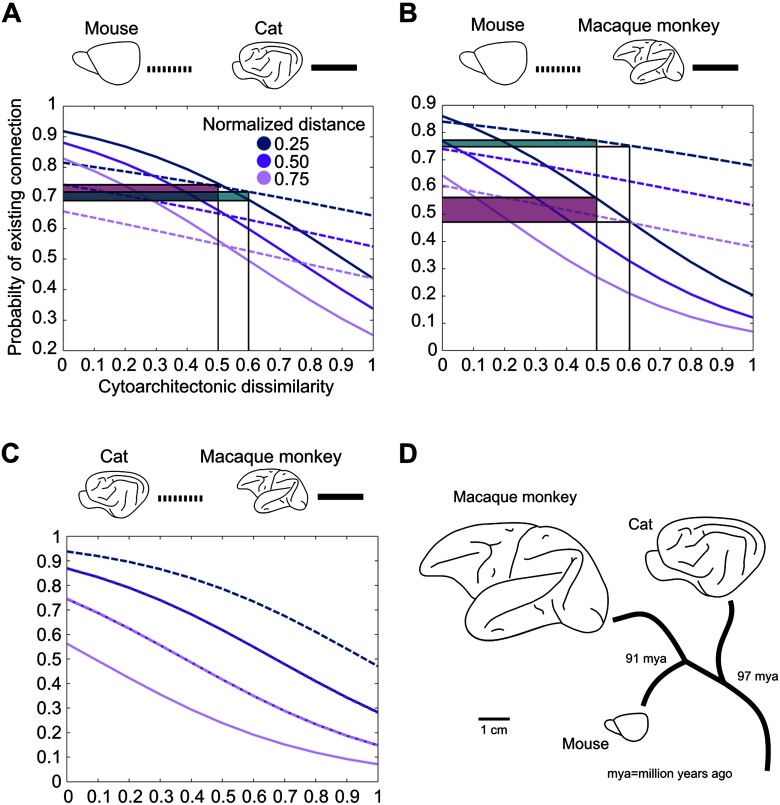
Cytoarchitectonic similarity relates to the existence of connections in a species-specific manner. (A) Increasing cytoarchitectonic dissimilarity of cortical areas results in a decrease of the probability of the existence of a connection. This decrease is more pronounced for the cat when compared with the mouse, as indicated by the larger probability decrease (shaded areas) for the same increase of cytoarchitectonic dissimilarity. (B) Same relation as in (A), but for the comparison of mouse versus macaque monkey. The shaded areas highlight the differences of probability of existence of a connection with an increase of cytoarchitectonic dissimilarity in the different species. Note that the illustrated differences of probability of existence can be visually demonstrated in other intervals, such as 0.4–0.5 or 0.7–0.8. The decrease of the probability of the existence of a connection is more pronounced for the macaque monkey when compared with the mouse. (C) Same relation as in (A), but for the comparison of cat versus macaque monkey. In this comparison, no species-specific differences of the effect of cytoarchitectonic similarity on the probability of connections were observed. (D) Brain size and phylogenetic distance of the mouse, macaque monkey, and cat. Adapted from Goulas et al. (2019).

Importantly, if differences in cortical architecture are to be predictive for connectivity features, such as the existence and laminar profiles of projections, there need to exist architectonic differences between the cortical territories in the first place. Consequently, in species such as rodents that show less pronounced differences between different cortical regions, a less pronounced alignment between cortical architecture and connectivity is expected. Indeed Goulas et al. (2019) showed that the cytoarchitectonic similarity of cortical areas relates to the existence of cortical connections in a species-specific manner (Figure 3). A species-specific relation between cytoarchitecture and connectivity is also apparent at the level of the global network topology of mammalian connectomes. Core areas, that is, areas tightly interconnected among themselves as well as with the rest of the brain, differ in terms of cytoarchitecture in relation to peripheral areas (less interconnected areas) in the cat and macaque monkey, with core areas also constituting the less differentiated and less neuronally dense areas (Figure 2, lower panels). However, this cytoarchitectonic segregation of core and periphery areas is statistically absent in the marmoset monkey and completely absent in the mouse. Cytoarchitecture varies systematically with other cytological features, such as spine density, and overall myelination. Thus, the synergy between topological segregation (core vs. periphery) and cytological properties or its absence across species might have species-specific functional implications and denote differences in the degree of vulnerability to pathology of these cortical areas across species (see Goulas et al., 2019 for details).

## INTEGRATION OF INTRINSIC CORTICAL ARCHITECTURE AND CIRCUITS WITH MACROSCOPIC CONNECTIONS

Cortical type indicates the intrinsic cytoarchitectonic organization of cortical areas as well as patterns of extrinsic, cortico-cortical connections of the areas. Moreover, the organization of area-intrinsic cortical circuits varies with type. In particular, eulaminate areas, with a well-defined laminar structure and high neuronal density, feature an intricate intrinsic circuitry that has been described as a canonical microcircuit (Binzegger, Douglas, & Martin, 2009; Douglas, Martin, & Whitteridge, 1989; Douglas & Martin, 2004; Potjans & Diesmann, 2012), in which intra- as well as interlaminar excitation are well balanced by populations of excitatory and inhibitory neurons. Despite the idea that this circuit may form a constant template across the cortical sheet, there exist variations of this template. Particularly, limbic (agranular and dysgranular) areas, which are characterized by the absence or a less marked appearance of a granular layer and generally show fewer apparent layers and a lower overall neuronal density, comprise a reduced microcircuit, which particularly appears to possess reduced interlaminar inhibition (Beul & Hilgetag, 2015).

**Figure 4.**
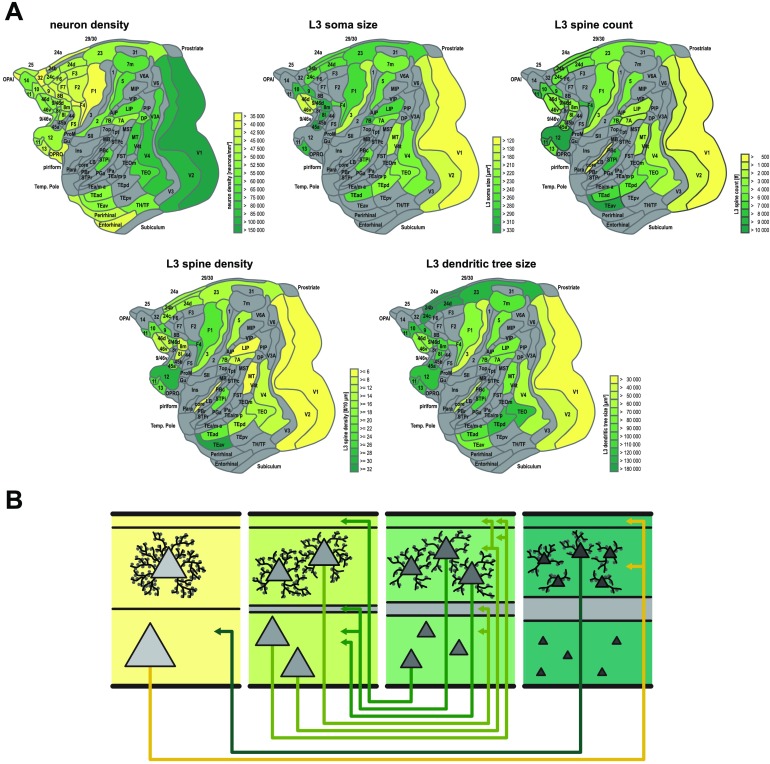
Integration of cortical macro- and microarchitecture with cortical connections. Less architectonically differentiated, agranular, cortical areas (yellow) are characterized by lower neuron density and different morphology of layer III pyramidal cells than more strongly differentiated, eulaminate, areas (dark green), with gradual changes across the spectrum. (A) Macroscopic and microscopic architectonic features show *concerted changes* along spatial cortical gradients, indicating a natural axis of cortical organization. In particular, higher neuron density tends to correlate with smaller cross sections of the soma and the dendritic tree as well as with lower total spine count and lower peak spine density. (B) Relations of architectonic types with connection features. Within cortical areas, the ratio of supra- versus infragranular soma size of projection neurons tends to increase as one transitions from less to more differentiated areas (externopyramidization; Goulas et al., 2018). Also note that projection neurons are displayed with relatively larger soma cross section than nonprojection neurons in the same cortical area and layer. Importantly, connections exist predominantly between areas of similar cortical type, and agranular and dysgranular regions (yellow) tend to form more connections than eulaminate regions (dark green). Hence agranular and dysgranular regions tend to be part of the network core, while eulaminate regions tend to be part of the network periphery (cf. Figure 2). Moreover, laminar patterns of projection origins are related to differences in architectonic differentiation. Connections between areas of distinct differentiation show a skewed unilaminar projection pattern, with projections originating predominantly in the infragranular or supragranular layers depending on the direction of the projection (agranular to eulaminate projections and eulaminate to agranular projections, respectively), while connections between areas of similar architectonic differentiation show a bilaminar projection origin pattern (connections between middle panels), where the dominating laminar compartment again depends on the connected areas’ relative differentiation. In sum, there are concurrent changes of macro- and microstructural cellular and connectional features across the cortical sheet, forming spatially ordered gradients, confirming and expanding observations from classic neuroanatomy studies (gradation principle of Sanides, 1962). Panels adapted from Beul and Hilgetag (2019).

Moreover, the findings described above demonstrate that several of the macroscopic and intrinsic structural features of cortical areas are linked. For example, higher neuronal density goes along with smaller cell cross sections and less elaborate morphological dendritic features, while lower density coincides with larger cell cross sections and more elaborate dendritic morphology (Beul & Hilgetag, 2019). The relations of connection features with the intrinsic connectional and structural organization of areas offer an opportunity for integrating microscopic and macroscopic architecture and connection features of cortical areas. These features are summarized in Figure 4. In terms of interareal connectivity, more frequent and denser connections exist between areas that are similar in cortical type, with similar overall neuron density. The projections originate in a bilaminar fashion from the upper and deep cortical layers of the source area, but predominantly in the upper cortical layers of the higher type areas, and they terminate across all layers of the target area, but predominantly in the middle layers (granular layer IV, where it exists) of the lower type areas. By contrast, areas of markedly different type are either not connected or only sparsely connected. Here, the projections arise mostly in a unilaminar fashion from the upper cortical layers of the source area and terminate on the middle to deep layers of the lower type target area. Such projections are complemented by projections from the deep layers of the lower type area that terminate in the upper layers of the higher type area.

Along with the regularities between cortical type or neuronal density and local morphological features, such as arborization of dendritic trees, these organizational principles of microscopic connectivity integrate microscopic cortico-cortical connections with the intrinsic circuits of each cortical area. Such generic rules can be used to inform particularly the development of large-scale cortical models. A first example of such a model has already been constructed (see “Implications of the Architectonic Type Principle for Large-Scale Simulations of Cortical Dynamics” herein; Schmidt, Bakker, Hilgetag, et al., 2018; Schmidt, Bakker, Shen, et al., 2018).

## DEVELOPMENTAL UNDERPINNINGS

Both within and across mammalian species, systematic covariation of multiple features of cellular morphology has been observed. This includes a higher number and higher density of spines and more complex dendritic arbors in prefrontal cortices of nonhuman primates (Bianchi et al., 2013; Elston, 2003, 2007, 2011) and higher total dendritic length, dendritic spine density, and dendritic spine numbers in the prefrontal cortex of the human brain (Jacobs, 2001). Moreover, it has been observed that with increasing soma size, the amount of heterochromatin in the nucleus decreases, while axon length and size of nucleus and nucleolus increase (García-Cabezas, Barbas, & Zikopoulos, 2018). Such gradual changes in cell morphology are aligned with the overall degree of architectonic differentiation across cortical areas. In the human brain, this systematic architectonic variation observed across the cortex has been directly linked to the timing of development (Barbas & García-Cabezas, 2016). Across species, differences in spine head size, spine neck length, and spine density have been reported between mouse and human cortex (Benavides-Piccione, Ballesteros-Yáñez, DeFelipe, & Yuste, 2002). More generally, a successive increase in dendritic complexity has been reported from New World monkeys to Old World monkeys to hominids (Bianchi et al., 2013; Elston, 2003, 2007; reviewed in Charvet & Finlay, 2014). These observations are consistent with an overall increase in neuron density as the length of developmental time schedules and brain size increase (reviewed in Caviness, Bhide, & Nowakowski, 2008, and Charvet & Finlay, 2014). To summarize, many features of cellular morphology seem to be tightly interlinked, corresponding to the overall degree of architectonic differentiation as well as differences in developmental timing. Together with this precise orchestration of cell specification during ontogenesis, which results in morphological features of neurons being attuned to the architectonic differentiation of an area as a whole, also the development of cortical connections appears to be closely coupled to architectonic differentiation. In data from two tract-tracing studies probing the developmental time course of projections within the visual cortex of the macaque monkey (Batardiere et al., 2002; Kennedy, Bullier, & Dehay, 1989), it can be observed that, even in the immature (prenatal or neonate) cortex, the fraction of projection neurons originating in supragranular layers is correlated with the difference in architectonic differentiation (as indicated by cortical type or neuronal density) between connected areas, and that immature laminar patterns of projection origins strongly correlate with eventual adult levels of supragranular contribution (Beul et al., unpublished observation). These observations indicate that the architectonic type principle successfully predicts the laminar origins of projections even at early stages of brain development. Therefore, basic ontogenetic mechanisms likely underlie its emergence.

**Figure 5.**
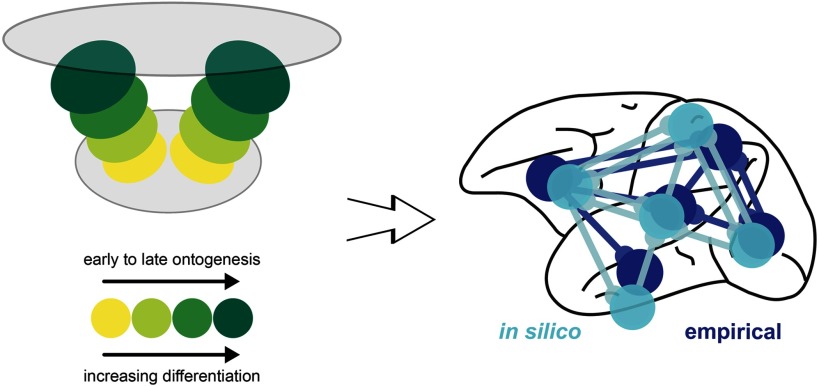
Developmental origins of the architectonic type principle. Summary of computational modeling of the ontogenetic development of cortical architecture and connections (Beul et al., 2018). The simulations indicate that the presence of two spatial origins of neurogenesis, resulting in two neurogenetic (temporal) and architectonic gradients, is necessary for the close correspondence of the *in silico* model to the empirical relations between connectivity and architectonic differentiation. Importantly, the empirically observed relations are replicated *in silico* only if the less-to-more differentiated architectonic gradients align with early-to-late ontogenetic gradients. Hence, the suggested mechanism is consistent with correspondence of time of neurogenesis to architectonic differentiation (e.g., Dombrowski, Hilgetag, & Barbas, 2001) and a dual origin of the cerebral cortex (Pandya, Seltzer, Petrides, & Cipolloni, 2014; Sanides, 1962).

In recent simulation experiments (Beul, Goulas, & Hilgetag, 2018), we explored whether spatiotemporal interactions in the forming cortical sheet could lead to the empirically observed connectivity consistent with the architectonic type principle. In an *in silico* model of cortical sheet growth and the concurrent formation of cortico-cortical connections, we systematically varied the spatiotemporal trajectory of neurogenesis and the relation between architectonic differentiation and time of origin of neural populations. We showed that, for realistic assumptions about neurogenesis, successive tissue growth and stochastic connection formation interacted to produce realistic cortico-cortical connectivity (Figure 5). The implication is that precise targeting of interareal connection terminations is not necessary to produce connectivity that resembles real brain networks within a cortical hemisphere. Using classifiers trained on such simulated cortico-cortical connection networks consistent with the architectonic type principle, we could successfully predict empirically observed connectivity in two species, cat and macaque. In similar simulations (Goulas, Betzel, & Hilgetag, 2019), we also demonstrated that interactions of structured spatial gradients and developmental time windows during ontogeny can explain the widely known features of homophily (Betzel et al., 2016) and distance dependence of connection strength, as well as a host of empirically observed patterns of network topology of vertebrate and invertebrate nervous systems. In sum, we demonstrated a possible mechanism of how relative architectonic differentiation and central features of connectivity become linked during development through spatiotemporal interactions (Beul et al., 2018), which supports previously stated hypotheses about the mechanistic underpinnings of the architectonic type principle (Barbas, 1986; Barbas, 2015; Hilgetag et al., 2016).

## IMPLICATIONS OF THE ARCHITECTONIC TYPE PRINCIPLE FOR LARGE-SCALE SIMULATIONS OF CORTICAL DYNAMICS

**Figure 6.**
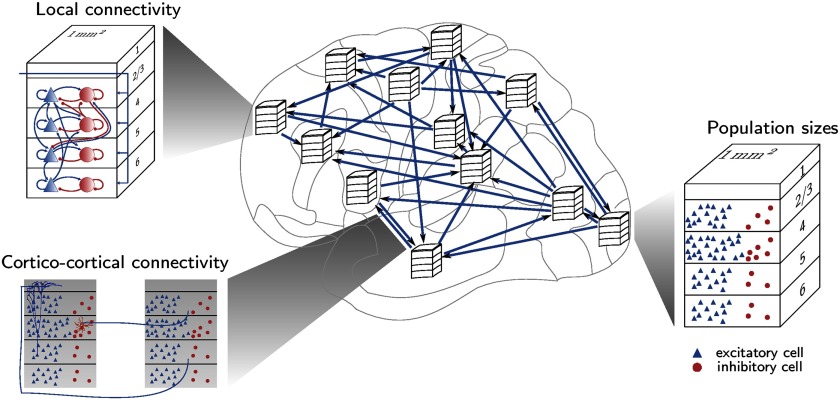
The architectonic type principle enables the generation of large-scale cortical models that integrate microscopic and macroscopic cortical architecture and connections. Schematic representation of a multiarea spiking model of macaque vision-related cortex, with laminar patterns of cortico-cortical connectivity determined in part from relative neuron densities of connected areas. Interarea and local connectivity together form polysynaptic pathways through the network. Figure reproduced from Schmidt, Bakker, Hilgetag, et al. (2018).

In addition to revealing wiring principles of the primate cerebral cortex, the exposed regularities of cortical structure can be employed to construct large-scale computational models of the cerebral cortex that are a unique tool for gaining novel insights into cortical dynamics and function (Figure 6). In particular, these regularities, including the systematic association of connectivity with cortical type or neuron density differences, enable connectomes underlying dynamical network simulations to be derived from incomplete experimental connectivity data. Schmidt, Bakker, Hilgetag, et al. (2018) used this approach, predicting laminar origin patterns of cortico-cortical connections from relative neuron densities of connected areas, to derive a layer-resolved connectivity matrix for all vision-related areas in one hemisphere of macaque cortex. Furthermore, the work exposed a close correlation between neuron density and cortical thickness, which was used to estimate missing thickness data and to help determine neural population sizes. As mentioned in the preceding, neurons in prefrontal cortices have a comparatively high number of dendritic spines in both humans and nonhuman primates (Bianchi et al., 2013; Elston, 2003, 2007, 2011; Jacobs, 2001). This feature is part of a more general upward gradient in the number of spines per neuron from high to low architectural types (Elston, 2002, 2003). The comparative constancy of the volume density of synapses across cortical areas (Harrison, Hof, & Wang, 2002) logically links the decrease in neuron density with the increase in the number of spines per neuron across areas of different architectonic type (Schmidt, Bakker, Hilgetag, et al., 2018). Thus, the gradient of architectonic types and associated morphological trends allow educated guesses for the full specification of cortical network models.

Such specification of complete connectivity graphs at the level of cortical areas and layers enables network simulations taking into account the corresponding detailed connectivity, as done by Schmidt, Bakker, Shen, et al. (2018) for all vision-related areas in one hemisphere of macaque cortex. This work studies resting-state activity in a network of interconnected microcircuits each with the full density of neurons and synapses, so that both the microscopic spiking activity and the macroscopic activity at the level of areas can be directly compared with experimental data. Good agreement with experimental observations was achieved simultaneously for microscopic and macroscopic activity at a metastable state of the network. The close correspondence with experimental activity data provides additional support for the underlying assumptions on the network structure. Such results, in conjunction with recent similar studies (e.g., Joglekar, Mejias, Yang, & Wang, 2018), showcase the need for computational models embodying the graded changes of micro- and macrostructure of cortex for more thoroughly explaining experimental observations at the functional level.

The fact that the architectonic type principle enables connectivity to be predicted not only at the level of cortical areas, but also at the laminar level, helps to identify polysynaptic pathways through the multiarea cortical network. A necessary step for this identification is to link cortico-cortical to intrinsic connectivity, by mapping cortico-cortical synapses to their target neurons and tracking the strongest pathways between areas that may pass through several intermediate populations within the same area. Following this approach, Schmidt, Bakker, Hilgetag, et al. (2018) found that the strongest paths between areas of similar type are like feedforward pathways in their start-to-end patterns, but like feedback pathways in terms of laminar patterns in intermediate areas.

The association of denser connectivity with lower architectonic types carries with it differences in spiking patterns of low- and high-type areas. In particular, Schmidt, Bakker, Shen, et al. (2018) found low-type areas to be more prone to bursting, which implies generally longer intrinsic time constants in line with a hierarchical organization of the width of single-neuron autocorrelation functions (Murray et al., 2014). This contribution of differential connection density to the hierarchy of intrinsic timescales may complement effects of area-specific receptor densities (Duarte, Seeholzer, Zilles, & Morrison, 2017).

Various studies have shown that a hierarchical separation of timescales at the level of neural populations or cortical areas matches nested frequencies present in the sensory environment and in motor behavior (Gordon, Koenig-Robert, Tsuchiya, van Boxtel, & Hohwy, 2017; Hasson, Yang, Vallines, Heeger, & Rubin, 2008; Kiebel, Daunizeau, & Friston, 2008; Victor & Purpura, 1996). These population- or area-level timescales mainly reflect correlations between neurons, rather than single-neuron autocorrelations, as the number of correlations grows with the square of the number of neurons (Hagen et al., 2016). Computational studies have linked the hierarchical trend in connection density (Chaudhuri, Knoblauch, Gariel, Kennedy, & Wang, 2015) or the topology of a rich-club core and less densely connected periphery (Gollo, Zalesky, Hutchison, van den Heuvel, & Breakspear, 2015) to hierarchically organized timescales, which in turn likely relate to layer-specific high-frequency feedforward and low-frequency feedback communication (Bastos et al., 2015; Mejias, Murray, Kennedy, & Wang, 2016; Michalareas et al., 2016; van Kerkoerle et al., 2014). However, these studies represent each area using coarse-grained equations, and thus do not distinguish between single-neuron and population-level dynamics, which can be markedly different. Direct comparison with the experimentally observed hierarchy of timescales in the sense of single-neuron autocorrelations (Murray et al., 2014) requires model predictions at the level of individual neurons. Furthermore, proper simultaneous predictions of single-neuron dynamics and pairwise cross-correlations in neural network models require using the full density of neurons and synapses (van Albada, Helias, & Diesmann, 2015). As shown by Schmidt, Bakker, Hilgetag, et al. (2018) and Schmidt, Bakker, Shen, et al. (2018), connectivity matrices informed by the architectonic type principle can help make such neuron-level predictions along with accurate predictions of large-scale dynamics.

Overall, relationships between cortical types and their connectivity inform various aspects of dynamic network simulations of the mammalian brain. For simulating the human brain, where invasive connectivity data are not available, predictive connectomics is inevitable in order to fully specify the network connectivity, implying even greater relevance of the architectonic type principle.

## CONCLUSIONS

Various aspects of macroscopic and microscopic cortical organization, such as architectonic type, cellular density, and size as well as dendritic size and spine density, are closely interrelated and present in spatially ordered gradients of cortical structure, defining a natural axis of cortical organization along which many macroscopic and microscopic cortical architectonic features covary. Moreover, these architectonic features are also related to the intrinsic cortical circuitry, as well as to features of the extrinsic, cortico-cortical connections. Thus, the architectonic type principle, which may derive from basic properties of spatially and temporally ordered cortical development, allows the integration of cortical architecture and connectivity across scales of organization, and provides specifications of multiscale models of cortical dynamics. Despite these intriguing findings; however, we are still at the beginning of understanding all the developmental, structural, and functional implications of this fundamental principle of cortical organization.

## ACKNOWLEDGMENTS

We thank Helen Barbas and Miguel A. García-Cabezas for helpful comments on the manuscript.

## AUTHOR CONTRIBUTIONS

Claus C. Hilgetag: Conceptualization; Funding acquisition; Investigation; Supervision; Writing – Original Draft; Writing – Review & Editing. Sarah F. Beul: Formal analysis; Investigation; Methodology; Software; Visualization; Writing – Review & Editing. Sacha J. van Albada: Conceptualization; Formal analysis; Funding acquisition; Investigation; Methodology; Software; Visualization; Writing – Review & Editing. Alexandros Goulas: Formal analysis; Funding acquisition; Investigation; Methodology; Writing – Review & Editing.

## FUNDING INFORMATION

Alexandros Goulas, Alexander von Humboldt Foundation, Humboldt Research Fellowship. Claus C. Hilgetag, Human Brain Project, Award ID: HBP/SGA2. Claus C. Hilgetag, German Research Council DFG, Award ID: SFB 936/A1. Claus C. Hilgetag, German Research Council DFG, Award ID: TRR 169/A2. Sacha J. van Albada, German Research Council DFG, Award ID: SPP 2041. Claus C. Hilgetag, German Research Council DFG, Award ID: SPP 2041, HI 1286/7-1.

## Note

^1^ “The thinkers however to whom we are referring attempt to state the nature of the soul only: with regard to the nature of the body which is to receive the soul they determine nothing in particular. And thus, although every body seems to possess a distinctive form and character, they act as if it were possible for any soul to cloth itself in any body…” (*Peri psychēs* I, 3).

## TECHNICAL TERMS

Laminar differentiation: The extent to which the cellular density and morphology of cortical layers differ and allow the layers to be discriminated.
Cortical type: An ordinal description and classification of cortical areas by the density and appearance of their cortical layers, ranging from less clearly differentiated agranular and dysgranular areas to more differentiated “eulaminate” areas (García-Cabezas et al., 2019; Hilgetag et al., 2016). Overall neuron density may be employed as a metric indicator of cortical type (Hilgetag, Medalla, Beul, & Barbas, 2016; Medalla & Barbas, 2006).
Architectonic type principle: The systematic relationships of essential cortical connection features to the cytoarchitecture of the mammalian cerebral cortex. These relationships manifest as variations of connectional features, such as existence or laminar origin and termination of projections, with spatially ordered, concerted variations of structural features, such as neuron density and cellular morphology, across the cortical sheet.
Externopyramidization: The rate of change of the ratio of soma size of supragranular versus infragranular pyramidal neurons. This rate can be calculated from the amount of change in soma size ratio across the cortical sheet.
